# Fully Automated, High-Dose Radiosynthesis of [^18^F]PARPi

**DOI:** 10.3390/ph15070865

**Published:** 2022-07-14

**Authors:** Anna Pacelli, Fadi Zarrad, Wolfgang P. Fendler, Ken Herrmann, Michael Nader

**Affiliations:** Klinik für Nuklearmedizin, Universitätsklinikum Essen (AöR), Hufelandstraße 55, D-45147 Essen, Germany; fadi.zarrad@uk-essen.de (F.Z.); wolfgang.fendler@uk-essen.de (W.P.F.); ken.herrmann@uk-essen.de (K.H.); michael.nader@uk-essen.de (M.N.)

**Keywords:** PET, fluorine-18, PARP, automation, radiosynthesis

## Abstract

[^18^F]PARPi is currently undergoing clinical trials as a PET tracer for many applications. However, only manual radiosynthesis was reported; this has several drawbacks, including an increased risk of contamination from the operator, and the need to limit the starting activity. The automation of the previously reported protocol for [^18^F]PARPi synthesis is challenging, as it requires transferring microvolumes of reagents, which many platforms cannot accommodate. We report a revised, high yield, and automated protocol for the radiosynthesis of [^18^F]PARPi, with final doses of over 20 GBq.

## 1. Introduction

Poly(ADP-ribose) polymerase (PARP) inhibitors are a novel class of chemotherapeutics, which have shown promise in the treatment of homologous recombination repair-deficient tumours [1,2]. Many clinical studies are investigating their use in monotherapy, as well as in combination with radio- and chemotherapy [3,4,5].

However, some patients are less likely to respond to PARP inhibitors due to low PARP expression, or to the development of resistance mechanisms [6]. The identification of responders versus non-responders using a non-invasive technique, such as positron emission tomography (PET), is a valuable approach to patient stratification. Therefore, several PET imaging tracers targeting PARP have been developed for this purpose [7,8,9,10].

**Scheme 1:**
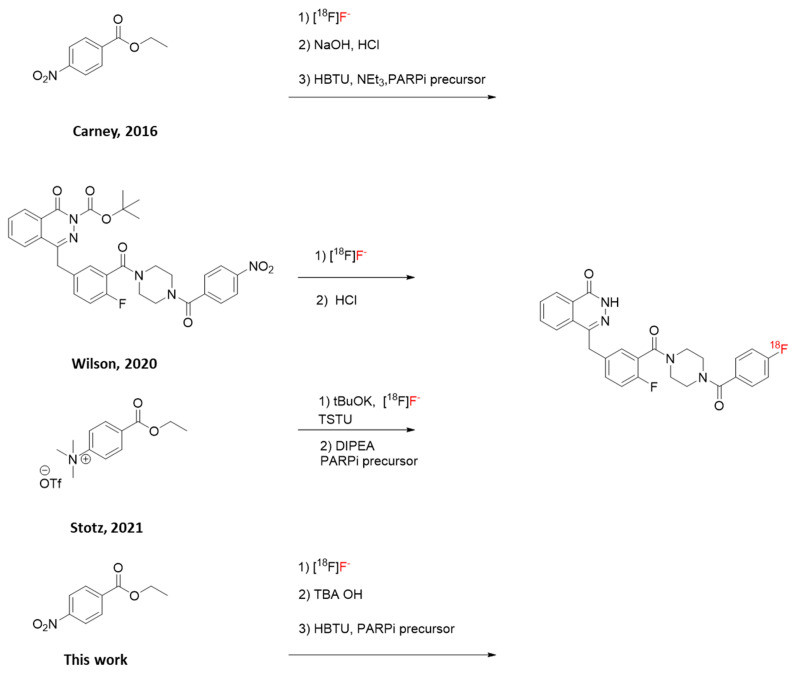
Radiosynthesis of [^18^F]PARPi with four different approaches [11,12,15].

**Scheme 2:**
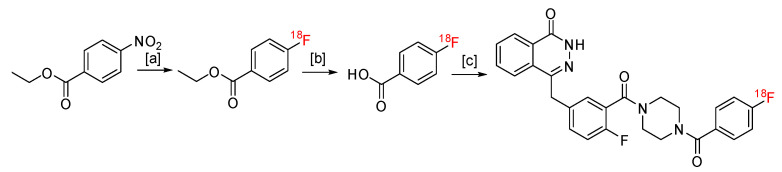
Reported synthesis of [^18^F]PARPi. [a]: [^18^F]fluoride, ethyl 4-nitrobenzoate (2 mg in 200 µL DMSO), 150 °C, 15 min; [b]: NaOH 1 M 0.5 mL, 1 min, RT, followed by HCl 1 M 0.5 mL; [c] PARPi precursor (4 mg in 100 µL DMSO), neat Net_3_ (25 µL), HBTU (10 mg in 100 µL DMSO) [11].

[^18^F]PARPi is a promising PET tracer for PARP imaging, and clinical trials have recently started to assess its application to oncological PET (NCT03631017 and NCT04173104; clinicaltrials.gov). However, the published three-step radiosynthesis (Scheme 1 and Scheme 2) showed low radiochemical yields (RCYs; 10% non-decay corrected, n.d.c.), both for the pre-clinical and for the clinical batches. The reported molar activity (A_m_) was also low (1.8 GBq/µmol). Neither automation nor the starting activity amounts were disclosed. The total synthesis time was 90 min [11].

An alternative, faster (66 min), two-step approach to [^18^F]PARPi has recently been published (Scheme 1); however, automation was not reported, and the n.d.c. RCY was 9.6%. Furthermore, the required precursor is, to our knowledge, not commercially available, and the highest starting activity used was 992 MBq [12].

Automation is a key feature in the routine preparation of the vast majority of PET tracers for clinical use, especially when labelled with ^18^F and ^11^C. An automated radiosynthesis can start at high radiation levels, as operators do not need to intervene throughout the procedure and are, therefore, protected from radiation exposure. Additionally, performing the procedure on a synthetic platform ensures reproducibility, and often results in shorter production time [13]. Finally, a limiting manual intervention reduces the risk of microbiological contamination; human operators are the most likely source of contamination in a GMP laboratory [7,14].

When we started working on this study, Stotz and co-workers published a novel two-step approach (Scheme 1), which was fully automated on GE Healthcare TRACERlab [15]. The required trimethylammonium precursor is commercially available, albeit less affordable than the precursor needed for the protocol reported by Carney and for this work. The total synthesis time was from 87 to 106 min. The A_m_ values ranged from 38.97 to 265.18 GBq/µmol. However, the reported RCYs were low (from 2.7% to 7.8% n.d.c.). The highest starting activity used was 64 GBq, an improvement over previously published methods.

In this study, we present the development of a revised protocol of the synthesis of [^18^F]PARPi reported by Carney and colleagues, leading to a fully automated, high-yielding, and high-dose production of this PET tracer.

## 2. Results and Discussion

The published protocol for the radiosynthesis of [^18^F]PARPi from ethyl 4-nitrobenzoate reports the use of small volumes that hinder automation (Scheme 2) [11]. Some of these volumes are comparable to the dead volume in a platform cassette, and in the case of triethylamine (Net_3_), are even lower. Therefore, initial efforts towards the feasibility of automation were focused on increasing the volumes of reagents. These tests were performed using a manual, one-pot approach.

**Figure 1:**
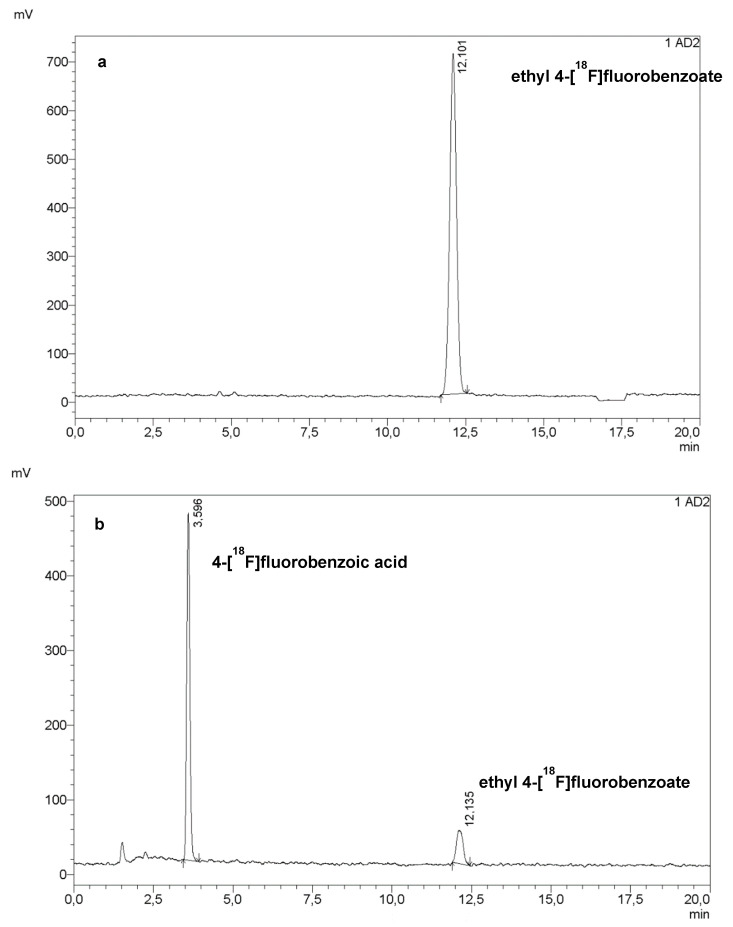
HPLC chromatogram of the crude mixture of the first step (**a**) and the second step (**b**) of the radiosynthesis of [^18^F]PARPi. Reaction conditions: (**a**) 2 mg PNEB in 500 µL dry DMSO, 15 min at 150 °C; (**b**) 0.5 mL of NaOH 1 M, RT, 1 min, followed by 0.5 mL of HCl 1 M.

The first step of the radiosynthesis of [^18^F]PARPi was the labelling of ethyl 4-nitrobenzoate (PNEB, 2 mg in 200 µL dry dimethyl sulfoxide, DMSO). The volume of DMSO was increased to 0.5 mL, then to 0.9 mL, with no detrimental effect to the radiochemical conversion (Figure 1a).

The following step was the hydrolysis of the ester to carboxylic acid using 0.5 mL of NaOH 1 M, which was then neutralised with 0.5 mL of HCl 1 M. At this stage, this step was performed as reported in the literature, resulting in an 85 to 95% hydrolysis of ethyl 4-[^18^F]fluorobenzoate. 

The coupling reaction was a challenging step, as the reported procedure required three small-volume solutions: 10 mg of (2-(1H-benzotriazol-1-yl)-1,1,3,3-tetramethyluronium hexafluorophosphate (HBTU) in 100 µL DMSO, 4 mg PARPi precursor in 100 µL DMSO, and 25 µL neat NEt_3_ (1 min at RT). The first approach was to target the HBTU and the precursor solutions. Table 1 lists all the conditions tested during this developmental phase. Unfortunately, these conditions yielded little to no final product.

**Figure 2:**
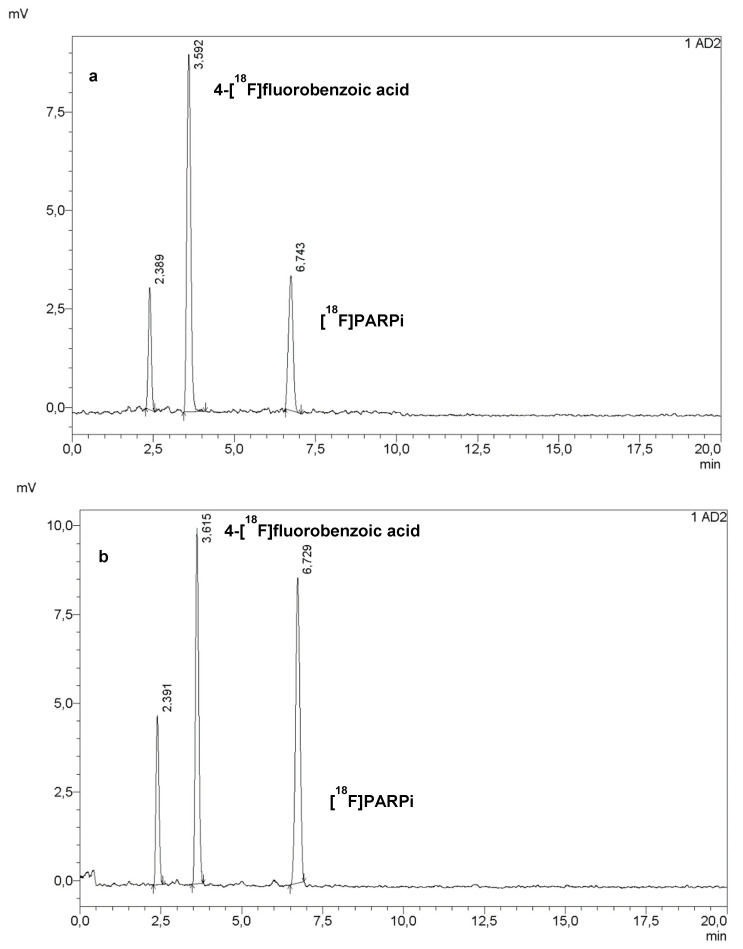
HPLC chromatograms of crude mixture from tests #6 (**a**) and #10 (**b**).

We decided to re-evaluate the conditions of the labelling reaction, based on the reasoning that, if more 4-[^18^F]fluorobenzoic acid was produced, then the coupling yield would increase. Therefore, the amount of PNEB was doubled; the hydrolysis conditions were left un-changed, and the coupling conditions chosen as a starting point were from test #4, which yielded slightly better results. As shown in Figure 2a, this resulted in a 27% radiochemical conversion (calculated via peak integration on HPLC chromatograms of crude mixtures). The further development of this step, mostly on the DMSO:water ratio, led to an increase in radiochemical conversion to 49% (Figure 2b). Table 2 lists all the conditions attempted during this phase.

Additionally, another coupling agent, 1-[bis(dimethylamino)methylene]-1H-1,2,3-triazolo[4,5-b]pyridinium 3-oxide hexafluorophosphate (HATU), was tested, but no product was observed; therefore, other coupling agents were not investigated.

We then moved on to optimising the addition of NEt_3_. When NEt_3_ was added to the precursor solution right before the addition of this mixture to the reaction vial, no yield decrease was observed. However, this test was not representative of an automated protocol, when solutions need to be prepared prior to the delivery of radioactivity; this can be up to one hour before the start of the synthesis, depending on its complexity. Therefore, we attempted adding NEt_3_ to other solutions one hour prior to the coupling reaction (Table 3). Unfortunately, all the conditions resulted in a decreased yield, from 26% (entry #17) to 6% (entry #14). When NEt_3_ was omitted, the coupling did not take place, suggesting that a tertiary base was necessary, as expected (entry #18) [16]. Additionally, no improvement was observed when NEt_3_ was diluted or added in larger amounts (entries #19 and #21), nor when the order of the addition of HBTU and the precursor was inverted (entry 20).

At this stage, we also started re-evaluating the conditions of the ester hydrolysis and the subsequent neutralisation. These steps required equal volumes of NaOH and HCl 1 M before the addition of the precursor. A strongly basic pH would deprotonate the phthalazin-1-one, while an acidic pH would protonate the piperazine, hindering the coupling step in both cases. Due to dead volumes in a cassette-based synthesiser, however, the addition of the exact same volumes might be not feasible. Therefore, we decided to test whether an organic base such as tetrabutylammonium (TBA) hydroxide could be used both for the hydrolysis and for the coupling, skipping, and, therefore, neutralisation step. This would potentially solve both the pH and NEt_3_ issues.

A preliminary test using 15 mg TBA hydroxide in 100 µL DMSO resulted in a 45% yield, comparable to the “standard” procedure with NaOH and NEt_3_ (as shown in the Supporting Information).

**Figure 3:**
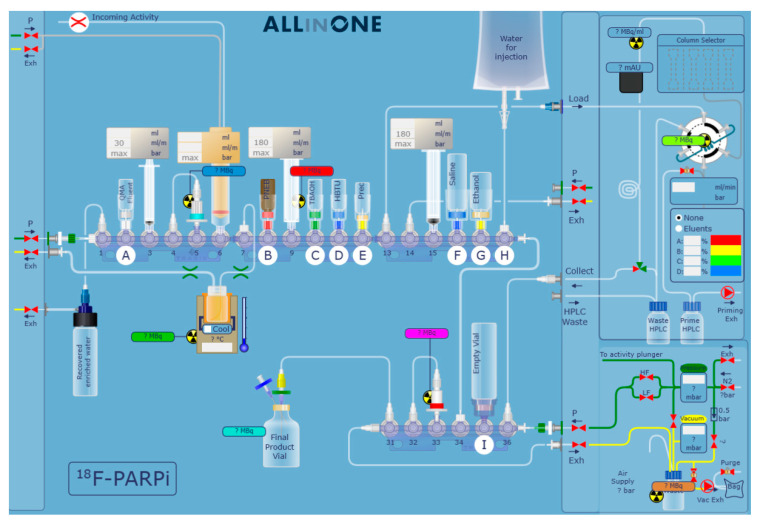
Automation scheme of [^18^F]PARPi synthesis with Trasis AllinOne.

**Figure 4:**
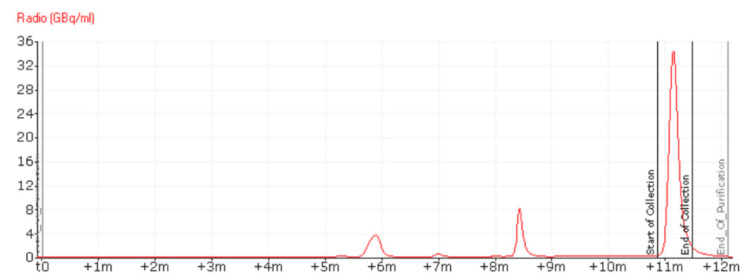
RadioHPLC chromatogram of the purification of [^18^F]PARPi via semi-preparative HPLC, using 35% MeCN in water + 0.5% acetic acid as eluent, in isocratic conditions. The peak at 8.5 min was identified as 4-[^18^F]fluorobenzoic acid. The peak at 6 min is an unknown impurity. The UV chromatogram of the semipreparative HPLC purification is available in the Supporting Information.

**Figure 5:**
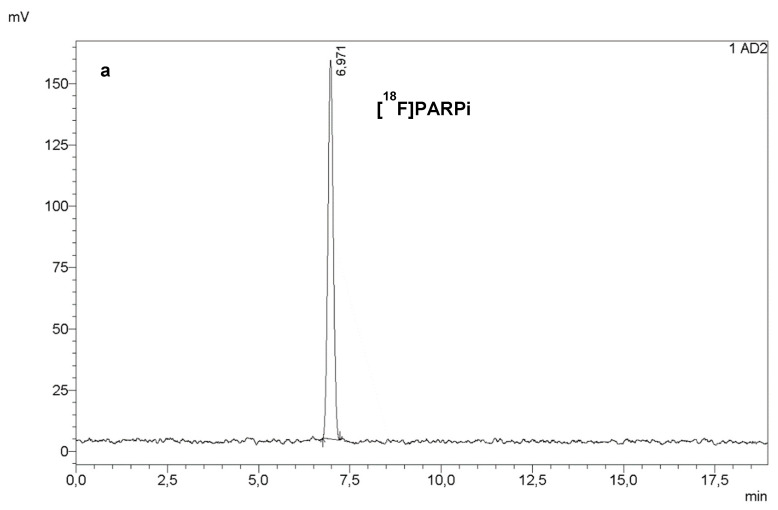

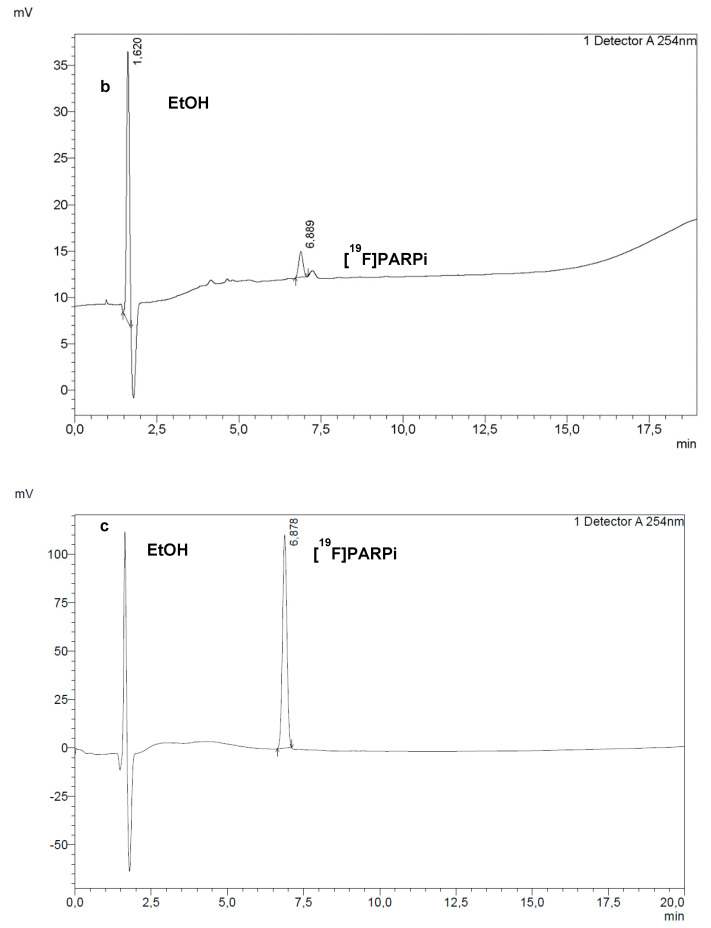
RadioHPLC (**a**) and UV HPLC at 254 nm (**b**) of a final dose of [^18^F]PARPi. Co-injection of a sample of final dose with a reference solution of [^19^F]PARPi (**c**) confirmed the identity of the PARPi peak in (**a**,**b**).

Having increased all volumes, we moved on to the development of the automation and to the optimisation of the semi-preparative HPLC purification. As shown in Figure 3, the synthesis was automated on a Trasis AllinOne platform equipped with a Knauer pump. The original protocol for the synthesis of [^18^F]PARPi requires a 35 min semi-preparative HPLC at 30% acetonitrile (MeCN) in water + 0.1% trifluoroacetic acid (TFA), likely to ensure that [^18^F]PARPi and nitro-PARPi would elute separately. We increased the percentage of MeCN to 35%, and swapped the 0.1% TFA with 0.5% acetic acid, due to its lower toxicity; we obtained a retention time of 11 min (Figure 4) with no detrimental effect on the chemical purity (Figure 5).

The reformulation process was kept as previously published; a tC18 cartridge was successfully used to remove the semi-preparative HPLC eluent and the product was reformulated in ≤10% ethanol (EtOH) in saline.

We performed the optimisation of the coupling step with TBA hydroxide and the scaling-up of the automated process simultaneously, starting with 2 to 5 GBq of [^18^F]fluoride. Increasing the amount of base to 30 mg (in 250 µL DMSO) resulted in a 15% RCY n.d.c. (calculated on the final, reformulated product). A further increase, as well as heating to high temperatures, was detrimental to the coupling. On the other hand, reducing the volumes of the HBTU and precursor solutions to 350 µL and heating at 40 °C had no major effect on the yield.

We then performed tests with 75–110 GBq of starting radioactivity to confirm the feasibility of the synthesis of a multi-patient dose. Serendipitously, we noticed that the radiosynthesis failed at the hydrolysis step when an anhydrous DMSO was used for the TBA hydroxide solution. We attributed this negative result to the necessity of water in order to increase the basicity of the TBA hydroxide, and performed a series of tests to optimise the percentage of water in DMSO.

Manual tests showed an excellent yield and complete conversion when 1% water was added (2.5 µL in 250 µL DMSO). Smaller amounts lowered the hydrolysis yield, and higher amounts favoured the formation of an unknown radioactive impurity.

However, in automated tests, the synthesis failed at the hydrolysis step when 1% water in DMSO was used. We, therefore, increased this percentage to 10%, obtaining an RCY of 20.5 ± 3.4% n.d.c. (calculated on the final, reformulated product), an A_m_ of 80.8±6.1 GBq/µmol, and a radiochemical purity of ≥97% (*n* = 6).

**Figure 6:**
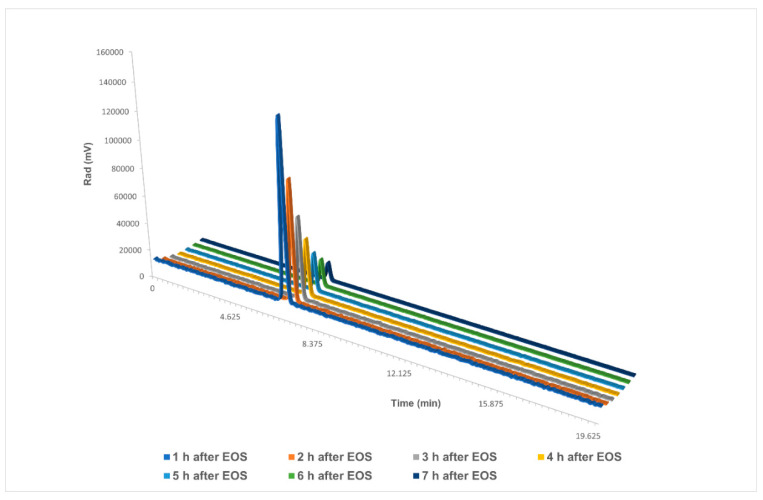
Stability analysis of [^18^F]PARPi over the course of 7 h in a final reformulation of ≤10% EtOH in saline. EOS: end of synthesis. Single chromatograms are available in the Supporting Information.

Additionally, we analysed the stability of the final dose at a high starting activity (Figure 6). Several HPLC analyses were run over the course of 7 h on a dose of 22.8 GBq, with obtained results starting from 110 GBq. The dose showed no decomposition in either the UV chromatogram or the radiochromatogram.

## 3. Materials and Methods

All chemicals were purchased from Sigma Aldrich except for PARPi precursor, reference standard, and nitro-analogue of the reference standard, which were kindly donated by Theragnostics, and used with no further purification.

No-carrier-added (n.c.a.) [^18^F]fluoride was produced with the (p,n) reaction of [^18^O]H_2_O in an IBA Cyclone 18/9 cyclotron.

Manual radiochemistry tests were performed on a Techne digital Dri-Block heater, using 5 mL glass V-vials. Approximately 350–500 MBq aliquots of [^18^F]fluoride were used for single tests. All conditions were tested at least in triplicates.

The optimised automated procedure was performed on a Trasis AllinOne module equipped with a Knauer pump, according to the following protocol:

[^18^F]fluoride (75–110 GBq) was trapped on a QMA carbonate Plus Light cartridge (130 mg sorbent per cartridge), which was previously conditioned with 5 mL of water for injection. [^18^F]fluoride was eluted with a solution containing TBA bicarbonate (2.8 mg) in water (100 µL) and MeCN (400 µL). After azeotropic drying, ethyl 4-nitrobenzoate (4 mg in 0.9 mL dry DMSO) was added to the reactor; the temperature was increased to 150 °C. After 15 min, the reactor was cooled down to 40 °C, and TBA hydroxide (30 mg in 250 µL DMSO plus 25 µL of water) was added. After 5 min, HBTU (10 mg in 350 µL DMSO) and precursor (8 mg in 350 µL DMSO) were added. After 5 min at 40 °C, the reaction mixture was diluted with water for injection, and injected into the semi-preparative HPLC column (Phenomenex Gemini 5 microns C6-Phenyl 250 × 10 mm). Purification was carried out in isocratic conditions (35 % MeCN in water+0.5% acetic acid), with a 5 mL/min flow. The product peak was collected at 11 min in a 50 mL vial filled with water. The diluted product was passed through a tC18 Plus Light cartridge (145 mg sorbent per cartridge), previously conditioned with EtOH (5 mL) and water (5 mL). The cartridge was rinsed with water (5 mL) and the product was eluted into a vial using EtOH (1.5 mL), passing through a Cathivex GV sterilising filter. Saline was then added to the final vial to yield a reformulation of ≤10% of EtOH in saline, with a final volume of 15 mL. The total synthesis time was 1 h 20 min.

Analytical HPLC analyses were performed on a Shimadzu Prominence UFPLC system. The column used for the analysis of the identity and (radio)chemical purity was a Phenomenex Kinetex biphenyl, 150 × 4.6 mm 5 microns, in isocratic conditions (35% MeCN in water + 0.1% TFA), at 1 mL/min, monitoring radiation and UV (254 nm). 

## 4. Conclusions

This study proposed a revised radiosynthesis of [^18^F]PARPi, with many advantages over the previously published methods, including a higher RCY, higher final dose, and high A_m_ [11,12,15].

A fully automated procedure on a cassette-based platform was developed and optimised, including a faster purification, with no detrimental effect on the purity of the final dose.

This approach is applicable to high starting activity (up to 110 GBq of [^18^F]fluoride, yielding over 20 GBq of the final dose), showing no radiolysis issues. Therefore, it is better suited for applications of [^18^F]PARPi imaging when high doses are needed, such as scanning multiple patients or delivering the tracer to other PET centres. 

## Figures and Tables

**Table 1:** Manual tests performed on the coupling step (*n* ≥ 3 for each entry). NEt_3_ kept as neat, 25 µL. Previous steps were performed as described in Figure 1. All solutions in DMSO. RCC: radiochemical conversion (average value), calculated via peak integration on HPLC chromatograms.

Entry	HBTU	Precursor	Reaction Conditions	RCC
1	10 mg in 0.5 mL	4 mg in 0.5 mL	1 min, RT	No product
2	10 mg in 0.5 mL	4 mg in 0.5 mL	10 min, RT	8.0% ± 1.5
3	10 mg in 0.5 mL	4 mg in 0.1 mL	1 min, RT	4.2% ± 0.4
4	10 mg in 0.5 mL	8 mg in 0.5 mL	1 min, RT	12.1% ± 1.7
5	10 mg in 0.5 mL	4 mg in 0.5 mL	5 min, 150 °C	6.0% ± 0.7

**Table 2:** Optimisation of DMSO:water ratio to increase the yield of the coupling step (*n* ≥ 3 for each entry). All non-specified conditions were kept as in entry #4. P: PARPi precursor.

Entry	Total DMSO (mL)	Total Water (mL)	Mass (mg)	RCC
6	1.5	1	4 PNEB, 8 P	27.1% ± 1.8
7	0.4	0.14	4 PNEB, 8 P	10.9% ± 2.9
8	1.5	0.5	2 PNEB, 4 P	8.9% ± 1.9
9	0.4	1	2 PNEB, 4 P	None
10	1.5	0.5	4 PNEB, 8 P	49.3% ± 6.5
11	1.5	0.5	4 PNEB, 4 P	None
12	1.5	0.5	2 PNEB, 4 P	15.1% ± 2.2

**Table 3:** Optimisation attempts of NEt_3_ addition for the coupling step (n≥3 for each entry). All other conditions were kept as entry #10.

Entry	NEt_3_ Addition	RCC
13	To precursor solution right before reaction	45.9% ± 3.6
14	To HBTU solution 1 h before reaction	5.9% ± 1.1
15	To NaOH solution 1 h before reaction	9.1% ± 1.0
16	To HCl solution 1 h before reaction	10.3% ± 2.7
17	To precursor solution 1 h before reaction	26.1% ± 3.1
18	None	None
19	Diluted in DMSO	7.0% ± 1.1
20	Diluted in DMSO, reagents addition swapped	6.9% ± 1.5
21	200 µL neat	14.3% ± 2.4

## Data Availability

Not applicable.

## Supplementary Materials

The following supporting information can be downloaded at: https://www.mdpi.com/article/10.3390/ph15070865/s1, Figures: single chromatograms of stability test; calibration curve for the calculation of the molar activity; radiochromatograms of the coupling step with NEt3 and with TBA hydroxide; semipreparative HPLC UV chromatogram at 220 nm.
