# Correction: Pacelli et al. Fully Automated, High-Dose Radiosynthesis of [18F]PARPi. *Pharmaceuticals* 2022, *15*, 865

**DOI:** 10.3390/ph16071024

**Published:** 2023-07-19

**Authors:** Anna Pacelli, Fadi Zarrad, Corentin Warnier, Thibault Gendron, Muhammad Otabashi, Charles Vriamont, Alex Jackson, Wolfgang P. Fendler, Ken Herrmann, Michael Nader

**Affiliations:** 1 Klinik für Nuklearmedizin, Universitätsklinikum Essen (AöR), Hufelandstraße 55, D-45147 Essen, Germany; fadi.zarrad@uk-essen.de (F.Z.); wolfgang.fendler@uk-essen.de (W.P.F.); ken.herrmann@uk-essen.de (K.H.); michael.nader@uk-essen.de (M.N.); 2 Trasis, Rue Gilles Magnee 90, 4430 Ans, Belgium; warnier@trasis.com (C.W.); gendron@trasis.com (T.G.); otabashi@trasis.com (M.O.); vriamont@trasis.com (C.V.); 3 Theragnostics Ltd., 1 Sans Walk, London EC1R 0LT, UK; alex.jackson@theragnostics.com

## Addition of Authors

Corentin Warnier, Thibault Gendron, Muhammad Otabashi, Charles Vriamont and Alex Jackson were not included as authors in the original publication [1]. The corrected Author Contributions statement appears here.

**Author Contributions:** A.P.: conceptualisation, investigation, original draft; F.Z.: review, conceptualization; C.W.: methodology, conceptualization; T.G.: methodology, conceptualization; M.O.: methodology, conceptualization, validation; C.V.: methodology, conceptualization; A.J.: funding acquisition, resources, conceptualisation; W.P.F.: funding acquisition, review; K.H.: funding acquisition, review; M.N.: review. All authors have read and agreed to the published version of the manuscript.

## Additional Affiliations

In the published publication, the following two affiliations are missing:

^2^ Trasis, Rue Gilles Magnee 90, 4430 Ans, Belgium

^3^ Theragnostics Ltd., 1 Sans Walk, London EC1R 0LT, UK

## Funding Correction

In the original publication, there was a mistake in Funding. The correct version should be:

**Funding:** This research was funded by Theragnostics Ltd.

## Conflicts of Interest Correction

A correction has been made to Conflicts of Interest. The updated text appears below.

**Conflicts of Interest:** Alex Jackson is an employee of Theragnostics Ltd. Charles Vriamont, Corentin Warnier, Thibault Gendron and Muhammad Otabashi are employees of Trasis.

The authors state that the scientific conclusions are unaffected. This correction was approved by the Academic Editor. The original publication has also been updated.
