# Supplementary material for: Fully Automated, High-Dose Radiosynthesis of [18F]PARPi
Source: Pharmaceuticals (Basel). 2022 Jul 14;15(7):865. doi: 10.3390/ph15070865 (PMC9317788; doi:10.3390/ph15070865)

pharmaceuticals

MDPI

Fully automated, high-dose radiosynthesis of [^{18}F]PARPi

Supporting Information

1. **Single chromatograms of [^{18}F]PARPi stability test**
2. **Calibration curve for the calculation of the molar activity**
3. **Radiochromatograms of the coupling step with NEt_3 and with TBA hydroxide**
4. **Semipreparative HPLC UV chromatogram at 220 nm**

1. Single chromatograms of [^{18}F]PARPi stability test

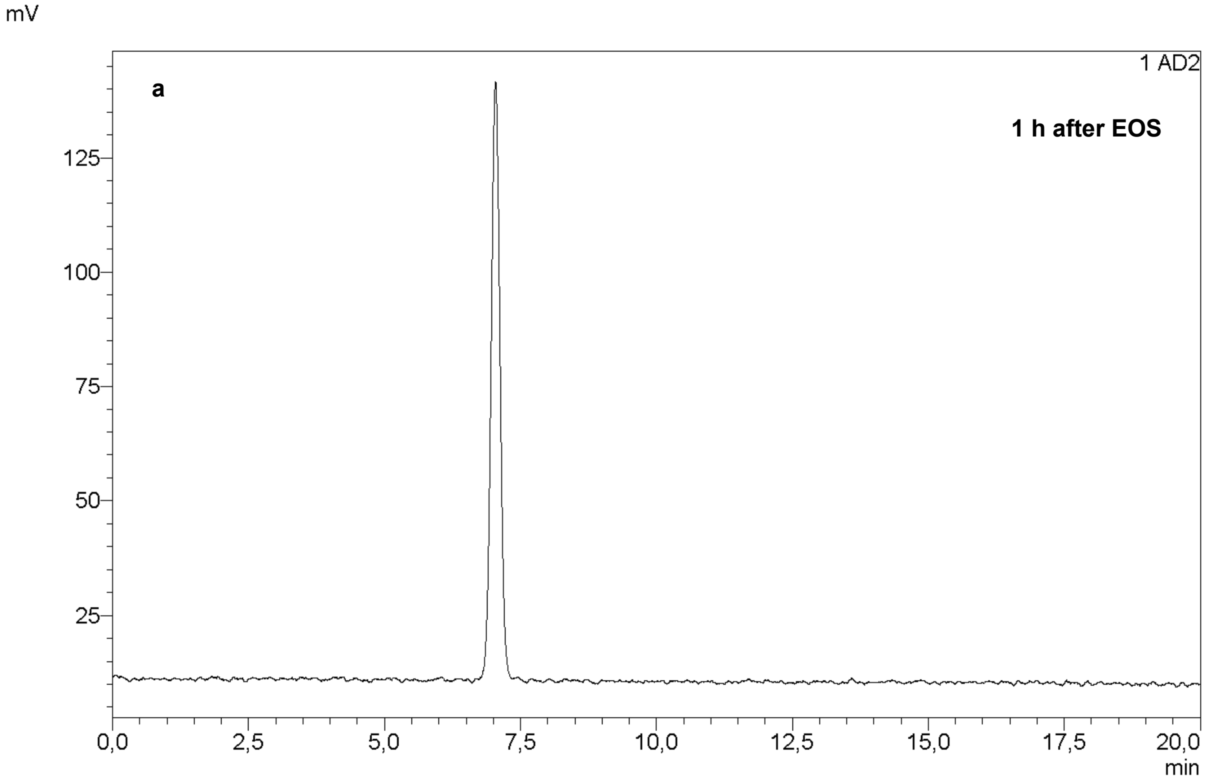

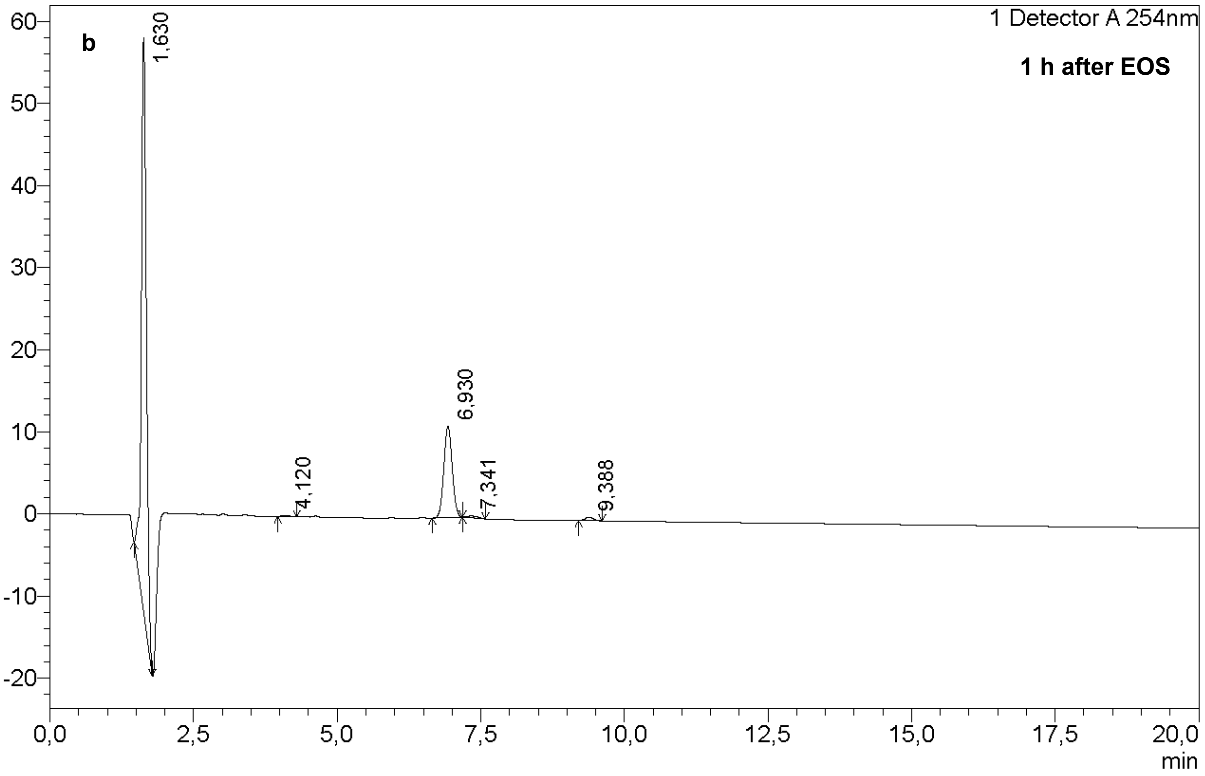

Figure S1. Radiochromatogram (**a**) and UV chromatogram (**b**) of [^{18}F]PARPi 1 hour after the end of the synthesis (EOS).

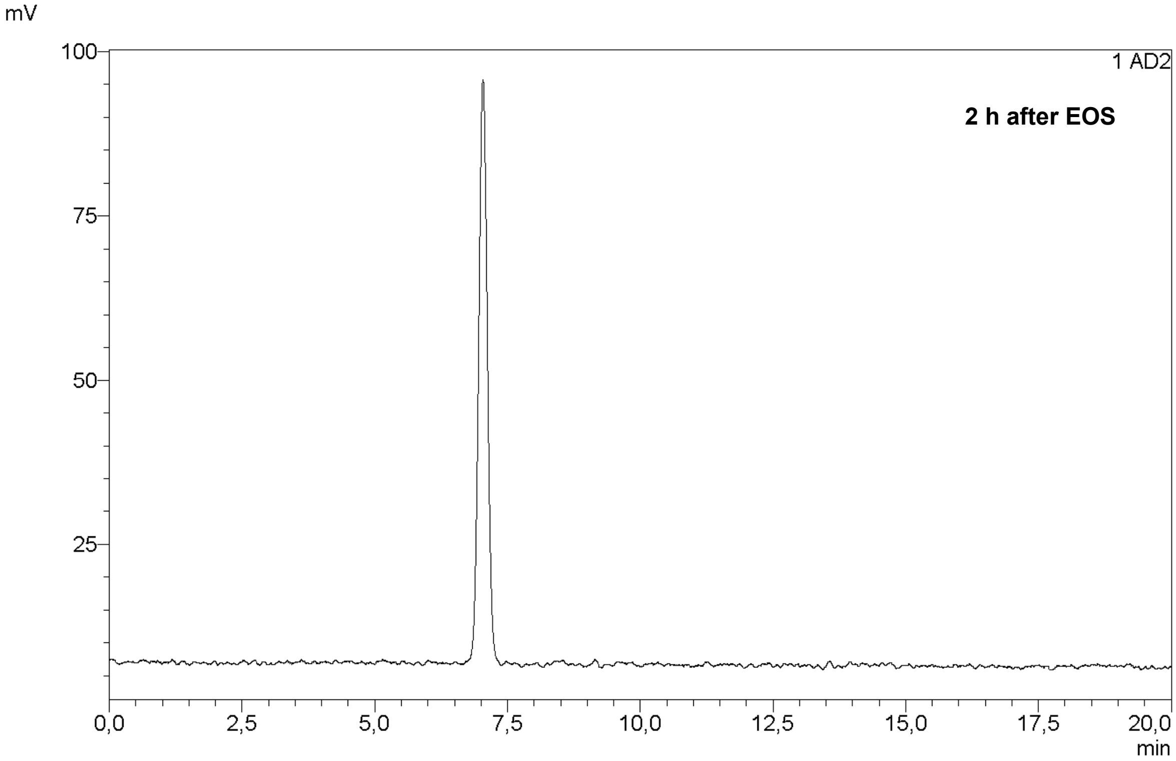

Figure S2. Radiochromatogram of [^{18}F]PARPi 2 hours after EOS.

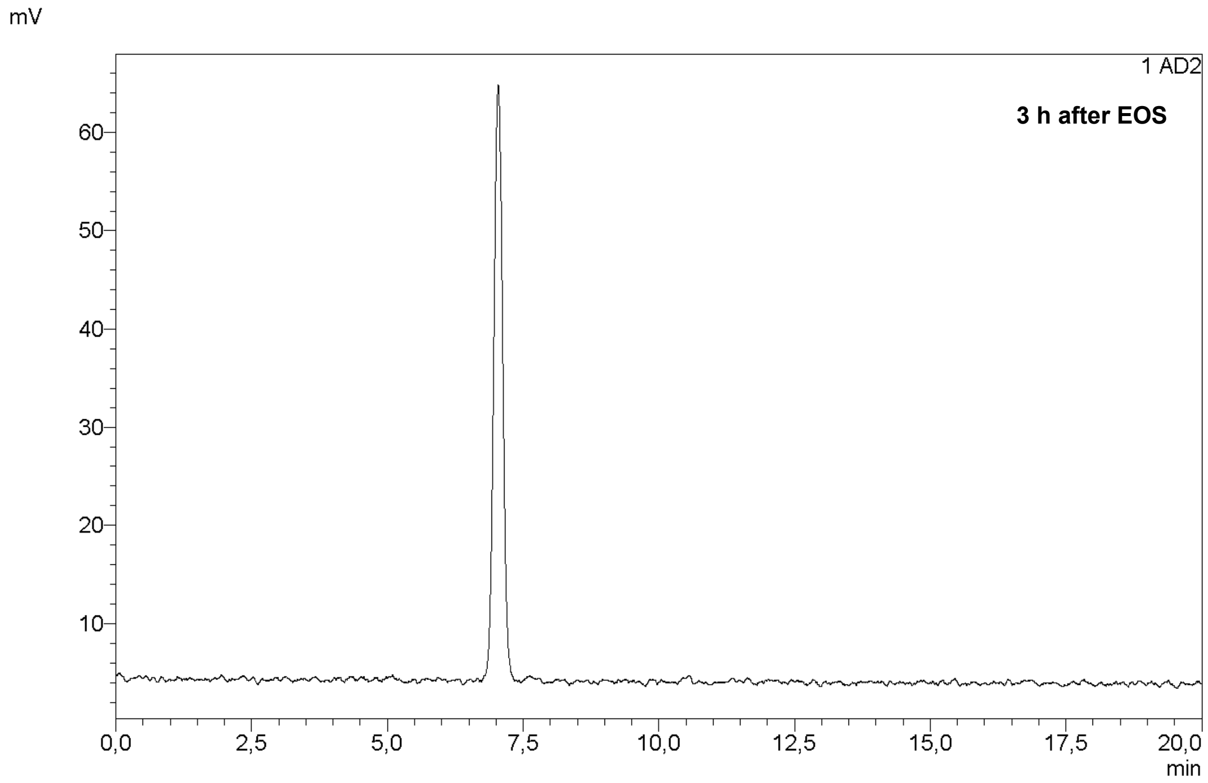

Figure S3. Radiochromatogram of [^{18}F]PARPi 3 hours after EOS.

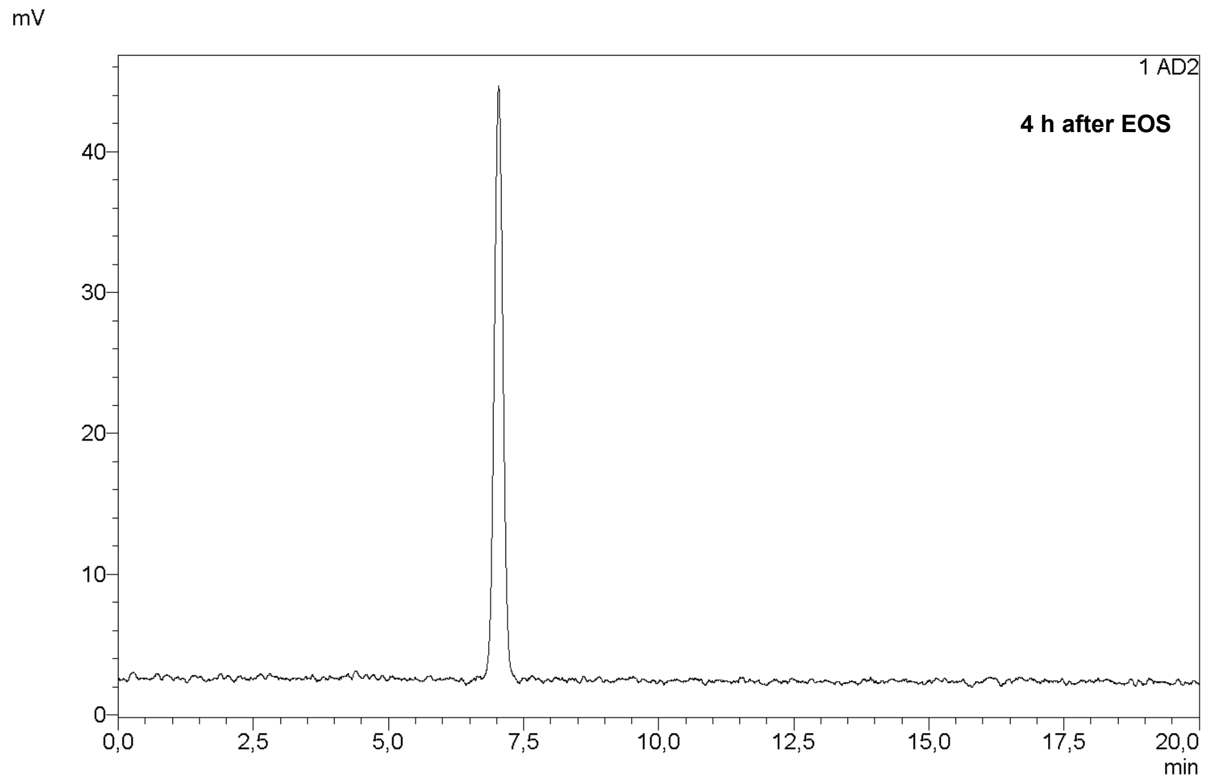

Figure S4. Radiochromatogram of [^{18}F]PARPi 4 hours after EOS.

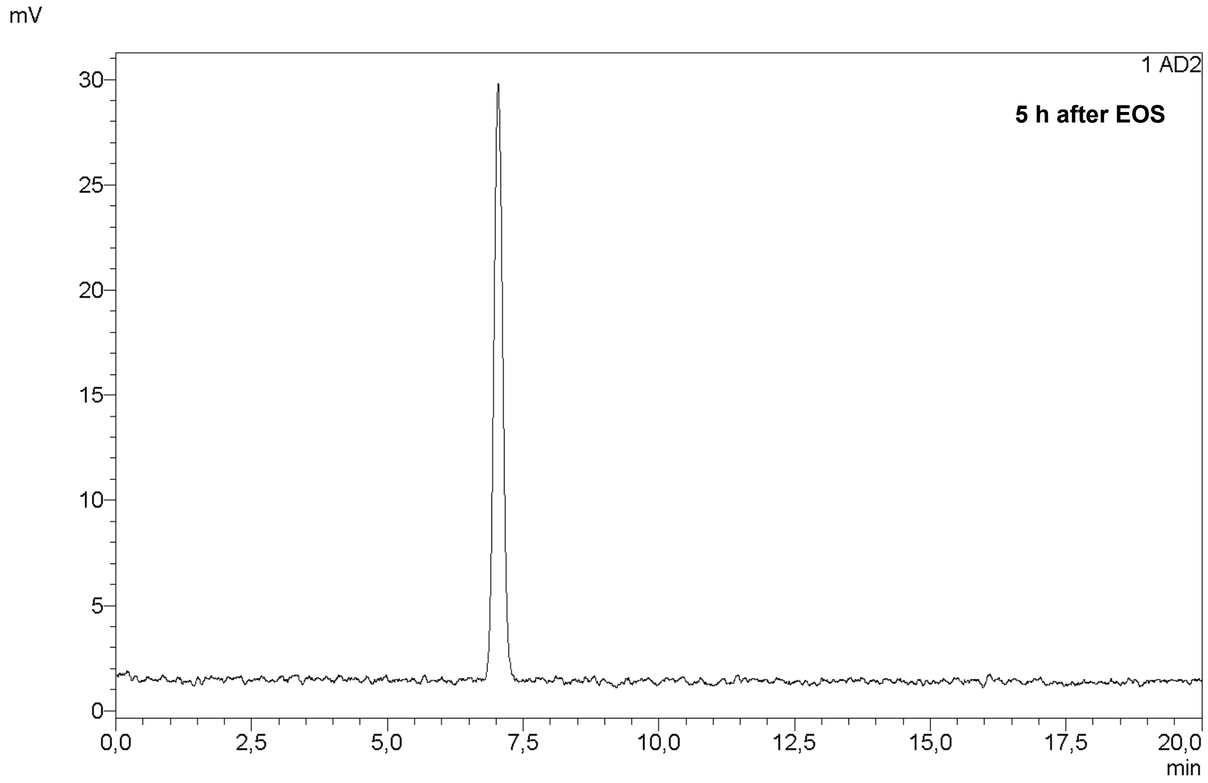

Figure S5. Radiochromatogram of [^{18}F]PARPi 5 hours after EOS.

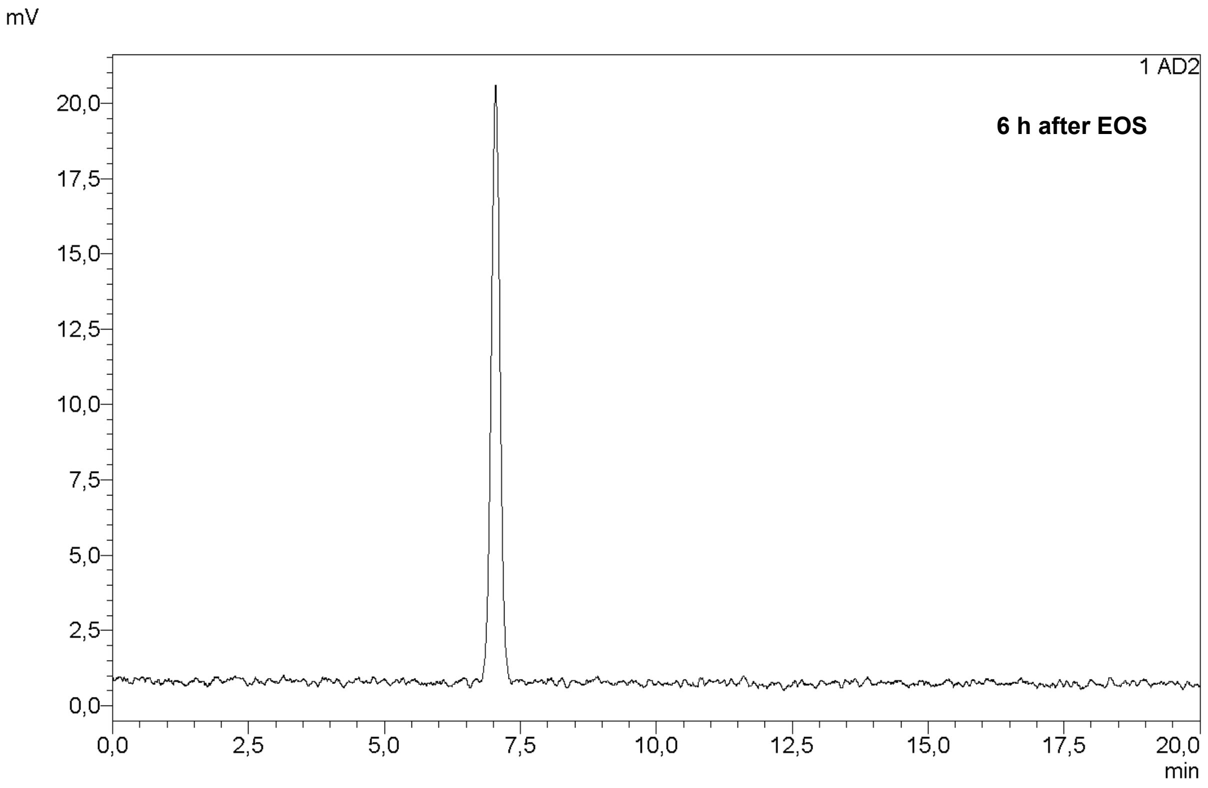

Figure S6. Radiochromatogram of [^{18}F]PARPi 6 hours after EOS.

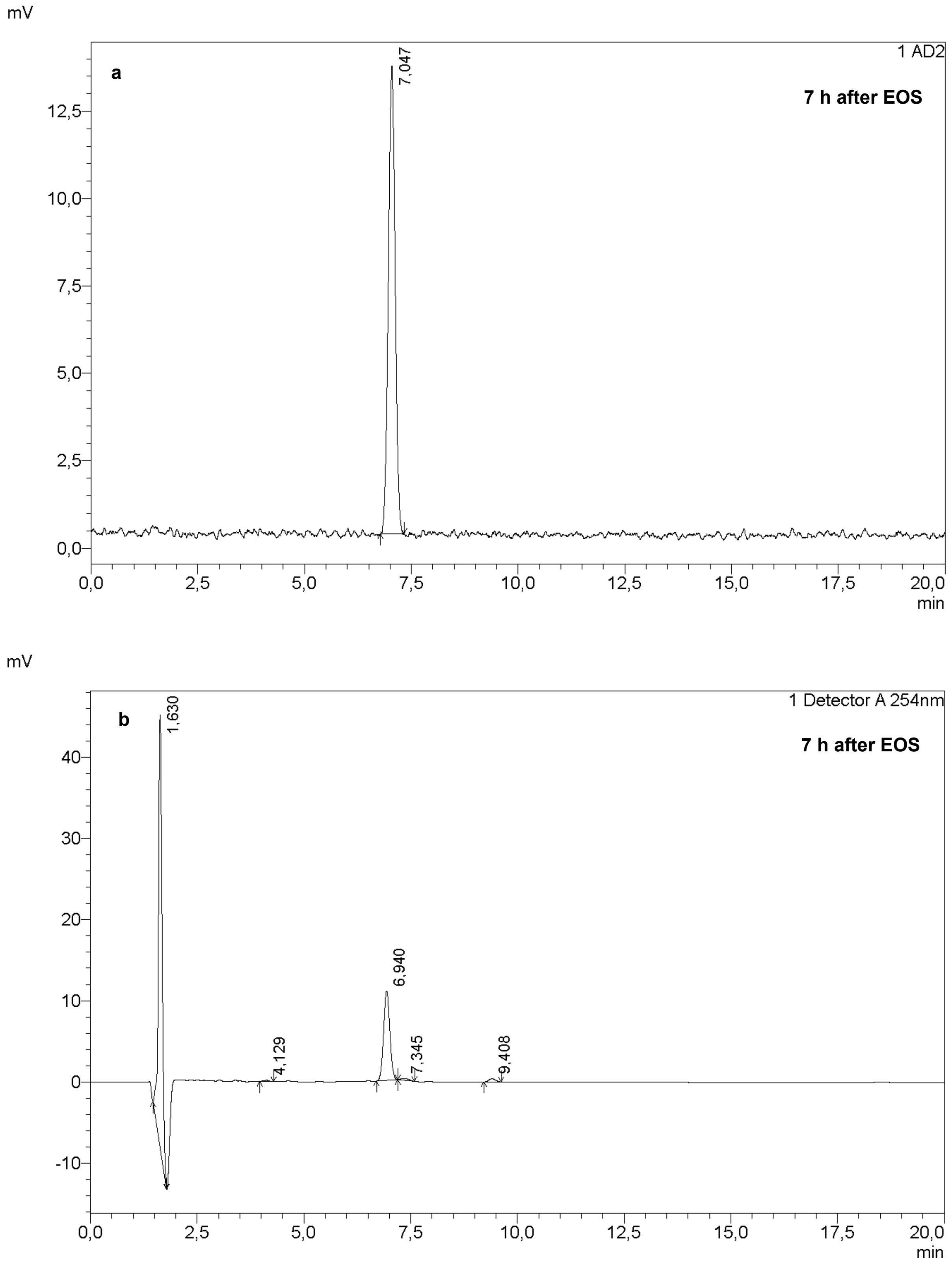

Figure S7. Radiochromatogram (**a**) and UV chromatogram (**b**) of [^{18}F]PARPi 7 hours after EOS.

2. Calibration curve for the calculation of the molar activity

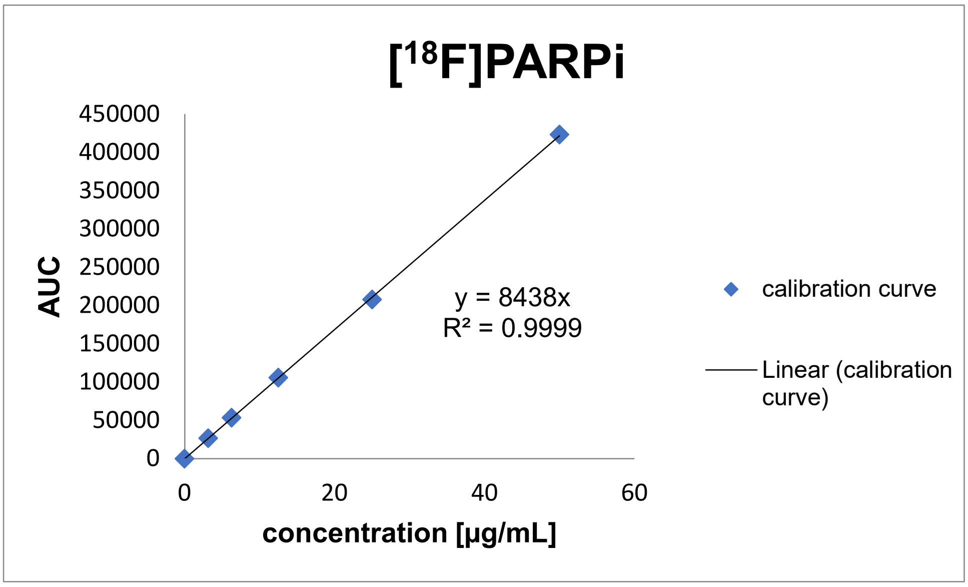

3. **Radiochromatograms of the coupling step with NEt_3 (a) and with TBA hydroxide (b)**

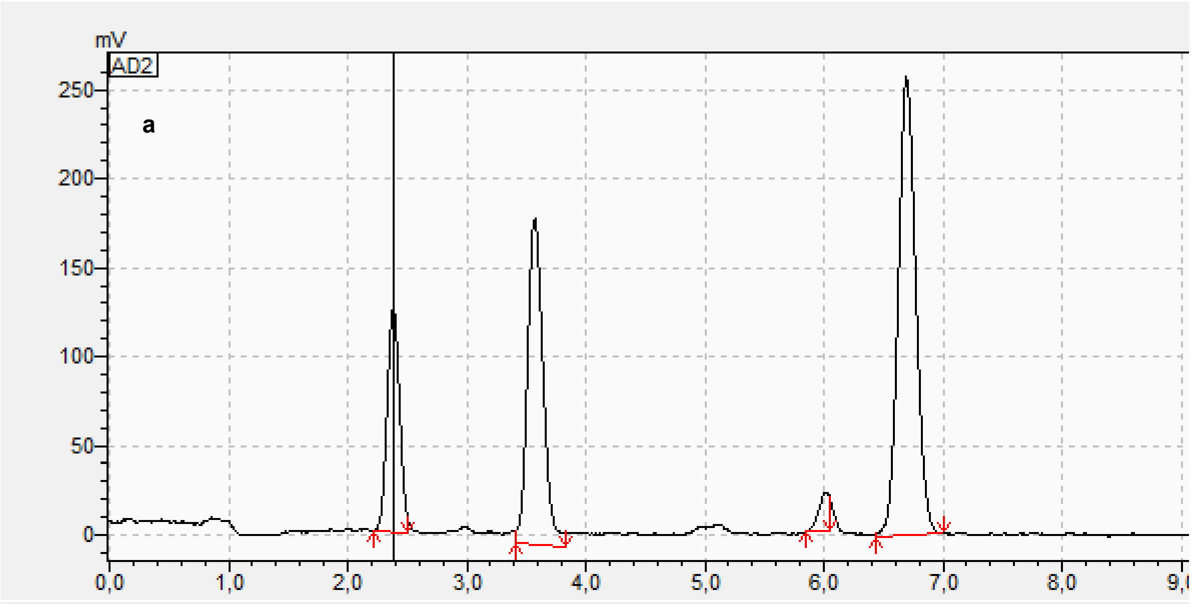

Results View - Peak Table

Peak Table | Compound | Group | Calibration Curve

Peak#	Ret. Time	Area	Height	Area%
1	2,387	912903	126329	17,152
2	3,572	1660059	183239	31,190
3	6,020	133350	21505	2,505
4	6,693	2613275	257045	49,100
5	19,817	2749	565	0,052
Total		5322335	588683	100,000

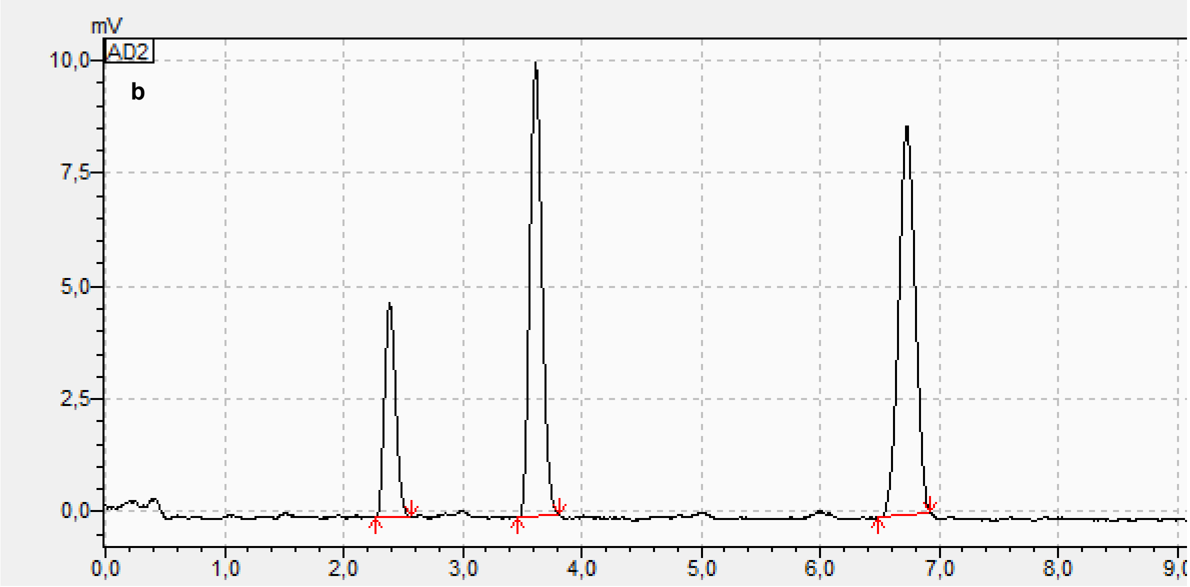

Results View - Peak Table

Peak Table | Compound | Group | Calibration Curve

Peak#	Ret. Time	Area	Height	Area%
1	2,391	30475	4769	16,603
2	3,615	70763	10030	38,552
3	6,729	82314	8617	44,845
Total		183553	23416	100,000

4. Semipreparative HPLC UV chromatogram at 220 nm

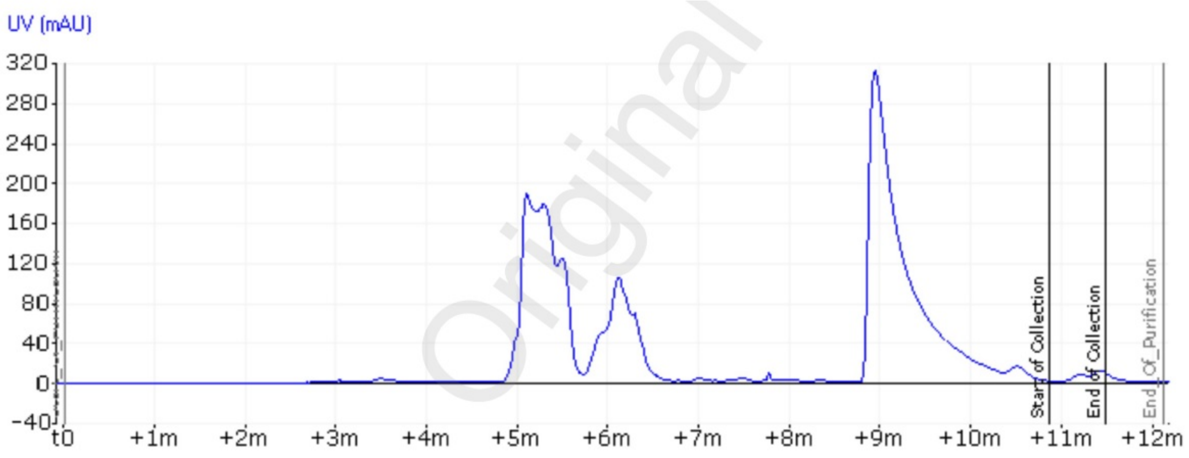

Supplement: Supplementary file 1 [file pharmaceuticals-15-00865-s001.zip › pharmaceuticals-1768541-SI.pdf]
